# The Dynamic Effects of Isosteviol on Insulin Secretion and Its Inability to Counteract the Impaired β-Cell Function during Gluco-, Lipo-, and Aminoacidotoxicity: Studies In Vitro

**DOI:** 10.3390/nu10020127

**Published:** 2018-01-26

**Authors:** Wenqian Gu, Andreas Rebsdorf, Kjeld Hermansen, Søren Gregersen, Per Bendix Jeppesen

**Affiliations:** Department of Endocrinology and Internal Medicine, Aarhus University Hospital, Tage-Hansens Gade 2, 8000 Aarhus C, Denmark; gu.wen.qian@clin.au.dk (W.G.); andreas.lund.rebsdorf@post.au.dk (A.R.); kjeld.hermansen@aarhus.rm.dk (K.H.); soeren.gregersen@aarhus.rm.dk (S.G.)

**Keywords:** isosteviol, steviol glycosides, insulin secretion, glucotoxicity, lipotoxicity, aminoacidotoxicity, mouse pancreatic islets, INS-1E cells

## Abstract

Isosteviol (ISV), a diterpene molecule, is an isomer of the backbone structure of a group of substances with proven antidiabetic capabilities. The aim of this study was to investigate if ISV elicits dynamic insulin release from pancreatic islets and concomitantly is able to ameliorate gluco-, lipo-, and aminoacidotoxicity in clonal β-cell line (INS-1E) in relation to cell viability and insulin secretion. Isolated mice islets placed into perifusion chambers were perifused with 3.3 mM and 16.7 mM glucose with/without 10^−7^ M ISV. INS-1E cells were incubated for 72 h with either 30 mM glucose, 1 mM palmitate or 10 mM leucine with or without 10^−7^ M ISV. Cell viability was evaluated with a Cytotoxic Fluoro-test and insulin secretion was measured in Krebs-Ringer Buffer at 3.3 mM and 16.7 mM glucose. In the presence of 3.3 mM glucose, 10^−7^ M ISV did not change basal insulin secretion from perifused islets. However, at a high glucose level of 16.7 mM, 10^−7^ M ISV elicited a 2.5-fold increase (−ISV: 109.92 ± 18.64 ng/mL vs. +ISV: 280.15 ± 34.97 ng/mL; *p* < 0.01). After 72 h gluco-, lipo-, or aminoacidotoxicity in INS-1E cells, ISV treatment did not significantly affect cell viability (glucotoxicity, −ISV: 19.23 ± 0.83%, +ISV: 18.41 ± 0.90%; lipotoxicity, −ISV: 70.46 ± 3.15%, +ISV: 65.38 ± 2.81%; aminoacidotoxicity: −ISV: 8.12 ± 0.63%; +ISV: 7.75 ± 0.38%, all nonsignificant). ISV did not improve impaired insulin secretion (glucotoxicity, −ISV: 52.22 ± 2.90 ng/mL, +ISV: 47.24 ± 3.61 ng/mL; lipotoxicity, −ISV: 19.94 ± 4.10 ng/mL, +ISV: 22.12 ± 3.94 ng/mL; aminoacidotoxicity: −ISV: 32.13 ± 1.00 ng/mL; +ISV: 30.61 ± 1.54 ng/mL, all nonsignificant). In conclusion, ISV acutely stimulates insulin secretion at high but not at low glucose concentrations. However, ISV did not counteract cell viability or cell dysfunction during gluco-, lipo-, or aminoacidotoxicity in INS-1E cells.

## 1. Introduction

Type 2 diabetes (T2D) continues to be a leading cause of death and mortality worldwide. It is characterized by hyperglycemia and frequently accompanied by hyperlipidemia and slightly elevated circulating amino acid levels. Inadequate levels of plasma insulin elevate hepatic glucose production, reduce insulin-mediated glucose uptake in skeletal muscle, and increase free fatty acid mobilization from adipose tissue, which promote the deterioration of glycemic control [1]. The total amount of released insulin in plasma depends on pancreatic β-cell proliferation and function [2], which plays a key role in T2D disease progression [3,4].

Chronic exposure to abnormally high blood glucose levels (glucotoxicity) promotes oxidative stress [5,6]. Subsequently, the adaptive antioxidant response impairs glucose-derived reactive oxygen species (ROS) signaling and glucose-stimulated insulin secretion (GSIS). Over time, this can perpetuate impaired pancreatic β-cell function and decreased β-cell mass [7,8]. Studies have reported that glucotoxicity negatively regulates insulin gene expression by decreasing insulin transcription factors, with pancreatic duodenal homeobox factor 1, BETA/NeuroD, and RIPE3b1/MafA included [5,9,10,11,12]. Kowluru et al. (2017) proposed that glucose toxicity induces inappropriate movement of the unprenylated yet constitutively active G protein Rac1, leading to β-cell apoptosis and dysfunction [13].

Prolonged exposure to high concentrations of palmitate has detrimental effects on β-cell viability and function [14,15,16,17,18], possibly mediated by endoplasmic reticulum stress [19], increased ROS [20,21], impaired mitochondrial functions [22,23], altered acetylation of multiple proteins [24]. We have previously shown that long-term exposure to high lipid concentrations (lipotoxicity) causes a series of alteration in pancreatic islets including relatively elevated glucagon secretion, decreased insulin secretion, loss of α-cell sensitivity to glucose, and an accumulation of triglycerides [25].

We have also demonstrated that chronic exposure to elevated levels of leucine and proline (aminoacidotoxicity) induces β-cell dysfunction, with increased basal insulin secretion and decreased GSIS in both isolated pancreatic islets and clonal β-cells [26,27,28]. Interestingly there is an association between high-protein intake and impaired glucose tolerance, insulin resistance, and T2D [29,30,31]. More over, during obesity and insulin resistance, high circulating levels of amino acids, e.g., leucine, proline, and valine, are seen [31]. 

Isosteviol (ISV) is mainly obtained by acid hydrolysis of stevioside, the sweet food additive extracted from the plant *Stevia Rebaudiana Bertoni* (Bertoni). Studies have shown that ISV possesses various biological activities including anti-hyperglycemic, anti-hypertensive, anti-tumor, anti-inflammatory, and antioxidant effects [32]. We have shown that ISV improves glucose and insulin sensitivity, lowers plasma triglycerides, lowers weight in diabetic KKAy mice, and markedly changes the gene expression profile of key insulin regulatory genes [33,34]. Additionally, we found evidence that ISV counteracts α-cell hypersecretion and contributes to changes in the expression of key genes after long-term exposure to palmitate [35].

In the present study, we tried to mimic T2D conditions in clonal β-cell line (INS-1E) by inducing gluco-, lipo-, or aminoacidotoxicity, and tested whether ISV could counteract the detrimental effects observed. We also wanted to investigate the dynamic insulin secretion elicited by ISV from pancreatic mouse islets. 

## 2. Materials and Methods

### 2.1. Materials

Tissue and cell culture medium RPMI 1640 was obtained from GIBCO BRL (Paisley, UK). Guinea pig anti-porcine insulin antibody, mono-^125^I-(Tyr A14)-labeled human insulin, and porcine insulin were from Novo Nordisk (Bagsvaerd, Denmark). Collagenase P was obtained from Boehringer Mannheim GmbH (Mannheim, Germany) and Hanks’ balanced salt solution (HBSS), bovine serum albumin (BSA), and other chemicals were obtained from Sigma Chemical (St. Louis, MO, USA). ISV was purchased from Wako Pure Chemical Industries (Tokyo, Japan) and was added to the medium from a stock solution (10^−2^ M) prepared in 99% ethanol.

50 mM palmitic acid: Palmitic acid (Sigma) was prepared by dissolving and heating equal molar amounts of NaOH, supplemented with distilled water, to obtain a concentration of 100 mM. It was further diluted with 10% BSA (fatty acid free) to 50 mM fatty acid, with 5% BSA. The stock solution was frozen at −20 °C until usage.

Modified Krebs-Ringer Buffer (M-KRB): 125 mM NaCl, 1.2 mM MgCl_2_, 5.9 mM KCl, 1.28 mM CaCl_2_, 25 mM 4-(2-hydroxyethyl)-1-piperazineethanesulfonic acid (HEPES), 5.0 mM NaHCO_3_ (pH 7.4; All Sigma). SYTO 24 solution: 5 mM SYTO 24 green fluorescent nucleic acid stain (molecular probes, Invitrogen, Eugene, OR, USA) in dimethyl sulfoxide was diluted to a final concentration of 0.01 mM. 

### 2.2. Isolation of Islets

Pancreatic islets were isolated from adult female NMRI mice (Taconic, Ry, Denmark) weighing 22 to 25 g by the collagenase digestion technique, as described previously [36,37]. Briefly, after the mice were anaesthetized with pentobarbital intraperitoneally, a midline laparotomy was applied and the distal end of the common bile duct was clamped at the papilla vateri. Thereafter, the hepatic duct was cannulated and 3 mL of ice-cold HBSS containing 0.3 mg/mL of Collagenase P was injected into the duct system of the pancreas. The whole pancreas was removed and then placed in a test tube in water bath at 37 °C for 19 min. After being washed three times with HBSS, the islets were hand-picked under a stereomicroscope and immediately transferred to RPMI 1640 medium and incubated overnight. Islets for perifusion studies were obtained from 12~20 mice to compensate for inter-individual differences. 

### 2.3. Perifusion of Islets

After overnight culture, the islets were rinsed twice with a M-KRB supplemented with 3.3 mM glucose and 0.1% BSA. In the perifusion experiments, 30 pre-incubated islets were transferred to each of the perifusion chambers [37]. The experiments were designed as follows: (1) 10-min pre-perifusion at 3.3 mM glucose; (2) 20-min perifusion at 3.3 mM glucose with/without ISV (10^−7^ M); (3) 40-min wash-out at 3.3 mM glucose; (4) 20-min perifusion at 16.7 mM glucose with/without ISV (10^−7^ M); (5) 40-min wash-out at 3.3 mM glucose; (6) 20-min perifusion at 16.7 mM glucose with 0.1 mmol/L carbamylcholine (Sigma). The flow rate was 75 μL/min. Samples were collected every 2 min.

### 2.4. Culture of INS-1E Cells

INS-1E cells (a generous gift from Prof. Claes B. Wollheim, Geneva, Switzerland) [38] with passage numbers between 70–89 were cultured in RPMI 1640 medium containing 11.1 mM d-glucose at 37 °C in a humidified atmosphere containing 95% air and 5% CO_2_. The medium was supplemented with: 10% fetal bovine serum, 100 IU/mL penicillin, 100 μg/mL streptomycin, 10 mM HEPES, and 5 μM 2-mercaptoethanol. The cells were passaged weekly. 

### 2.5. Viability of INS-1E Cells 

INS-1E cells were seeded in 96-well Black Visiplate TC plates (Wallac Oy, Turku, Finland) at a density of 3 × 10^4^ cells/well in 100 μL medium. The cells were allowed to adhere overnight. Thereafter, they were treated and cultured with different concentrations of glucose, palmitic acid, and leucine with/without 10^−7^ M ISV or 10^−7^ M Glucagon-like peptide-1 (GLP-1). After 72 h, the number of dead cells in each well was calculated using a fluorometric assasy kit based on the cell lysis and staining method (Cytotoxic Fluoro-test Wako; Wako Pure Chemical Industries, Osaka, Japan) in the FLUOstar Galaxy (BMG, Ramcon, Denmark).

### 2.6. Insulin Secretion from INS-1E Cells

The INS-1E cells were seeded in 24-well Black Visiplate TC (Wallac Oy, Turku, Finland) plates at a density of 3.0 × 10^5^ cells/well in 1 mL medium. After adhering overnight, the cells were cultured in RPMI 1640 with different concentrations of glucose, palmitic acid, and leucine with/without 10^−7^ M ISV or 10^−7^ M GLP-1. After 72 h of incubation, the cells were pre-incubated with M-KRB supplemented with 3.3 mM glucose and 0.1% BSA for 15 min and then the cells were incubated in 1 mL M-KRB containing 3.3 or 16.7 mM glucose for 1 h. Subsequently, supernatants (300 μL) were collected, centrifuged, and 200 μL were kept at −20 °C for insulin analysis. After the secretion study, the number of cells was estimated using nuclear staining with 0.01 mM SYTO 24 reagent (20 μL/well) and measured by FLUOstar Galaxy. Insulin levels were normalized to cell number.

### 2.7. Insulin Assay

Insulin was analyzed by radioimmunoassay using guinea pig anti-porcine insulin antibody (Novo Nordisk, Bagsvaerd, Denmark). Mono-^125^I-(Tyr A14)-labeled human insulin (Novo Nordisk) was used as tracer and rat insulin (Novo Nordisk) was used as a standard. Ethanol was added to separate bound and free radioactivity. The inter- and intra-assay variation coefficients were both less than 5%. 

## 3. Statistical Analysis

All data analysis was performed with GraphPad Prism Software Version 7.0 (GraphPad Software, San Diego, CA, USA). Statistical significance between two groups was evaluated using unpaired Student’s *t*-test. Data are presented as the mean ± standard error of the meam (SEM); *p*-values < 0.05 were considered significant.

## 4. Results

### 4.1. Effects of ISV on the Dynamic of Insulin Release from Perifused Mouse Islets

**Figure 1:**
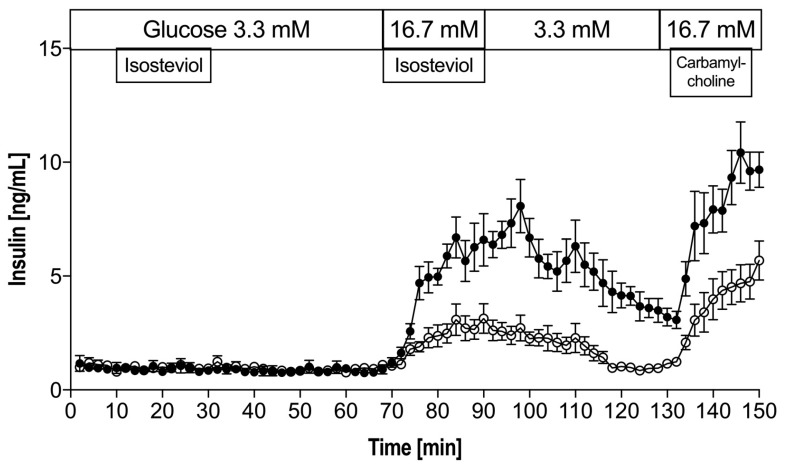
Insulin secretion from mouse islets in perifusion experiments in the absence (control ○) or presence (intervention ●) of 10^−7^ M isosteviol (ISV) at 3.3 mM and 16.7 mM glucose. Each curve represents the average ± standard error of the meam (SEM) of six perifusion experiments, each containing 30 islets. Experiments were finished off with carbamylcholine as a positive control at 16.7 mM glucose.

In the presence of 3.3 mM glucose, the addition of ISV did not change basal insulin secretion. As expected, a biphasic insulin response was found when glucose level was increased from 3.3 to 16.7 mM. Figure 1 shows that in the presence of high levels of glucose (16.7 mM), ISV (10^−7^ M) elicited a pronounced and sustained 2.5-fold (*p* = 0.0016) monophasic increase in insulin release. At 130–150 min, the insulin AUC (area under the curve) increased 2-fold (*p* = 0.0058) in the ISV group compared to the control.

### 4.2. Impact of Gluco-, Lipo-, and Aminoacidotoxicity on the Viability of INS-1E Cells 

#### 4.2.1. Glucotoxicity

**Figure 2:**
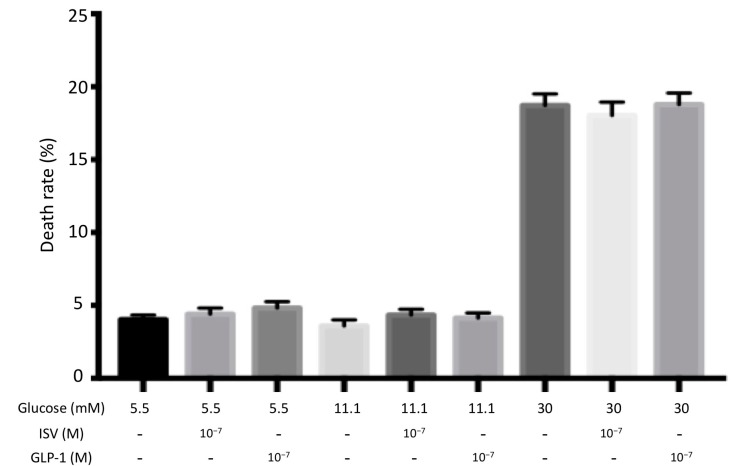
Effects 10^−7^ M ISV and 10^−7^ M Glucagon-like peptide-1 (GLP-1) on cell death rate in glucose-treated INS-1E cells. We measured cell death rate following 72 h of incubation with or without 10^−7^ M ISV/10^−7^ M GLP-1, in the presence of 5.5 mM, 11.1 mM, or 30 mM glucose. Data are presented as the mean ± SEM of 28 samples per group from three independent experiments.

INS-1E cells were challenged with low (5.5 mM) and high (30 mM) glucose for 72 h with and without 10^−7^ M ISV and 10^−7^ M GLP-1. A significant increase of cell death rate was discovered at high glucose levels compared to the control group (11.1 mM glucose), while no change was found at low glucose levels. However, no significant change was induced by co-incubation with either 10^−7^ M ISV or 10^−7^ M GLP-1(Figure 2).

#### 4.2.2. Lipotoxicity 

**Figure 3:**
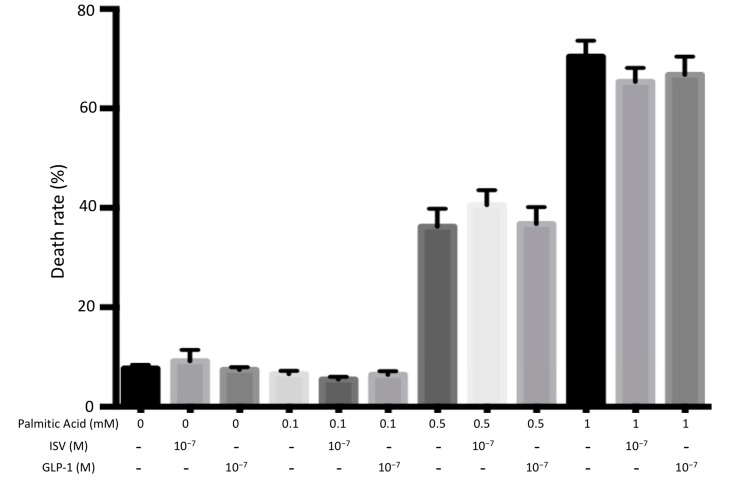
Effects 10^−7^ M ISV and 10^−7^ M GLP-1 on cell death rate in palmitic acid-treated INS-1E cells. We measured cell death rate following 72 h OF incubation with or without 10^−7^ M ISV and 10^−7^ M GLP-1, in the medium containing 0.1 mM, 0.5 mM, or 1 mM palmitic acid. Data are presented as the mean ± SEM of 28 samples per group from three independent expriments. The vehicle of 0 mM palmitic acid is equivalent to that of 0.5 mM palmitic acid.

Figure 3 shows the effect of 10^−7^ M ISV and 10^−7^ M GLP-1 on the viability of INS-1E cells treated with 0.1 mM, 0.5 Mm, or 1 mM palmitic acid. Cell death level was significantly increased to 36% at 0.5 mM palmitic acid, and to 70% at 1 mM palmitic acid. No significant difference was found after co-incubation with either 10^−7^ M ISV or 10^−7^ M GLP-1.

#### 4.2.3. Aminoacidotoxicity

**Figure 4:**
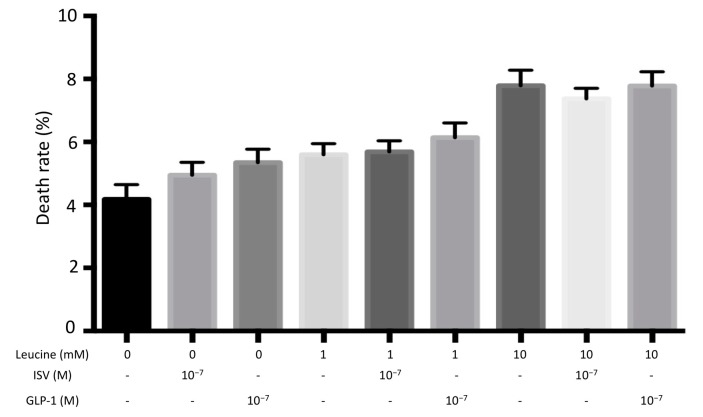
Effects of 10^−7^ M ISV and 10^−7^ M GLP-1 on cell death rate in leucine-treated INS-1E cells. We measured cell death rate following 72 h of incubation with or without 10^−7^ M ISV and 10^−7^ M GLP-1, in the medium containing 1 mM or 10 mM leucine. Data are presented as the mean ± SEM of 28 samples per group from three independent experiments.

As shown in Figure 4, cell death rates were slightly increased when INS-1E cells were exposed to 1 mM and 10 mM leucine. However, no significant change was induced by co-incubation with either 10^−7^ M ISV or 10^−7^ M GLP-1.

### 4.3. Impact of Gluco-, Lipo-, and Aminoacidotoxicity on Insulin Secretion of INS-1E Cells 

#### 4.3.1. Glucotoxicity

**Figure 5:**
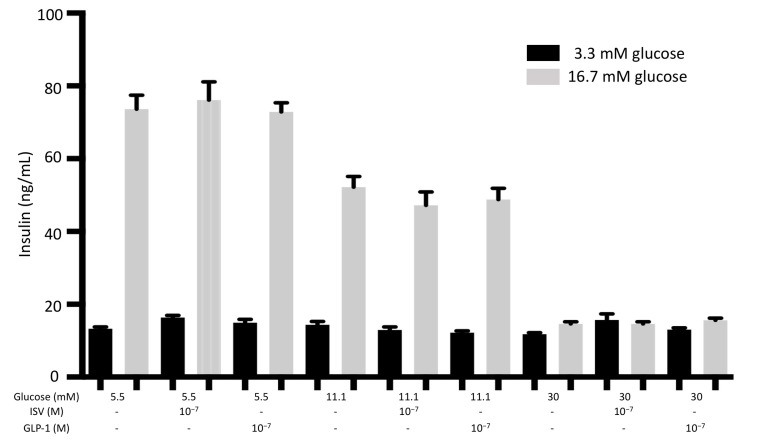
Effects of 10^−7^ M ISV and 10^−7^ M GLP-1 on insulin secretion from glucose-treated INS-1E cells. After 72 h of incubation with or without 10^−7^ M ISV and 10^−7^ M GLP-1, in the medium containing 5.5 mM, 11.1 mM, or 30 mM glucose, cells were stimulated with low (3.3 mM) and high (16.7 mM) glucose for 1 h, and subsequently insulin secretion was measured. Data are presented as the mean ± SEM of 18 samples per group from three independent experiments.

Figure 5 shows that at 3.3 mM glucose, BIS (basal insulin secretion) from INS-1E cells remained unchanged after 72 h of incubation with 5.5 mM and 30 mM glucose. High glucose (16.7 mM) stimulated insulin secretion increased after 72 h of exposure of the cells to 5.5 mM glucose, but decreased insulin secretion to 30 mM glucose. Neither 10^−7^ M ISV nor 10^−7^ M GLP-1 elicited any significant changes from INS-1E cells incubated at 5.5 mM, 11.1 mM, and 30 mM glucose

#### 4.3.2. Lipotoxicity

**Figure 6:**
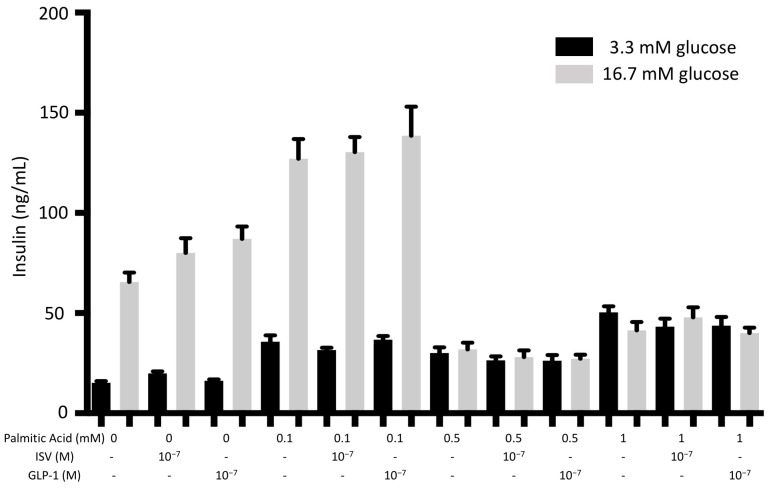
Effects of 10^−7^ M ISV and 10^−7^ M GLP-1 on insulin secretion in palmitic acid-treated INS-1E cells. After 72 h of incubation with or without 10^−7^ M ISV and 10^−7^ M GLP-1, in the medium containing 0.1 mM, 0.5 mM, or 1mM palmitic acid, cells were stimulated at low (3.3 mM) and high glucose (16.7 mM) for 1 h. Subsequently, insulin secretion was measured. Data are presented as the mean ± SEM of 18 samples per group from three independent experiments. The vehicle of 0 mM palmitic acid is equivalent to that of 0.5 mM palmitic acid.

As can be seen in Figure 6, there was no significant change in BIS at 3.3 mM glucose from INS-1E cells after 72 h of incubation with 0.1 mM, 0.5 mM, or 1 mM palmitic acid. By contrast, high concentrations (0.5 mM and 1 mM) of palmitic acid significantly decreased insulin secretion after 72 h. Neither 10^−7^ ISV nor 10^−7^ GLP-1 made a significant change in this situation.

#### 4.3.3. Aminoacidotoxicity

**Figure 7:**
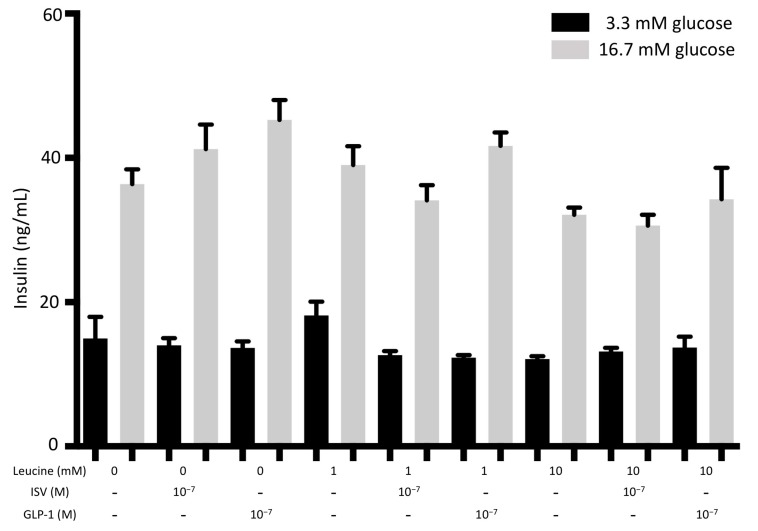
Effects of 10^−7^ M ISV and 10^−7^ M GLP-1 on insulin secretion in leucine-treated INS-1E cells. After 72 h of incubation with or without 10^−7^ M ISV and 10^−7^ M GLP-1, in the medium containing 1 mM or 10 mM palmitic acid, INS1-E cells were stimulated with low (3.3 mM) and high (16.7 mM) glucose. Subsequently, insulin secretion was measured. Data are presented as the mean ± SEM of 18 samples per group from three independent experiments.

When INS-1E cells were exposed to 1 mM or 10 mM leucine for 72 h, no significant effect on insulin secretion was found compared to the control group. Both 10^−7^ M ISV and 10^−7^ M GLP-1 showed no effect in these conditions, as illustrated in Figure 7.

## 5. Discussion

Numerous studies have shown that the major steviol glycosides, stevioside and Rebaudioside A, possess anti-hyperglycemic effects [39,40,41,42,43,44]. The stevioside derivative, ISV, has a higher bioavailability and a more potent insulinotropic effect. This study is the first to demonstrate that ISV causes a dynamic insulin stimulatory effect. However, ISV is not able to counteract the toxic effects of chronic exposure of INS-1E cells to high concentrations of glucose, palmitic acid, or leucine.

T2D is a chronic metabolic disorder that results from relative insulin deficiency and insulin resistance. In T2D patients, hypoglycemia is a major safety issue that can be fatal, particularly in patients with cardiovascular diseases. The risk of hypoglycemia is one of the main reasons preventing patients from achieving optimal glucose levels [45,46]. The incidence of hypoglycemia is a major drawback for sulfonylureas, a classic medication towards T2D, and it has therefore been assigned a lower priority in the AACE/ACE (American Association of Clinical Endocrinologists/American College of Endocrinology) treatment algorithm for T2D [47]. There is an urgent need to identify potential new drugs that enable T2D patients to both achieve glycemic goals and avoid hypoglycemia simultaneously. Steviol glycosides seem to have this potential. In the perifusion experiment, we have demonstrated that ISV elicits a distinct monophasic insulin response, similar to what we have previously found for stevioside, Rebaudioside A, and steviol [40,44]. That is, ISV stimulated insulin secretion in a dose-dependent manner. It showed no insulinotropic action at a low glucose level of 3.3 mM, while it caused a clear-cut insulin release at high glucose levels. Consequently, our results indicate that ISV possesses the desired potential in the treatment of T2D, since the insulinotropic action present at high glucose levels disappears at low glucose concentrations.

Carbamylcholine is a cholinergic agonist, which depolarizes the β-cell by the activation of the acetylcholine receptors. In the present study, carbamycholine is used as a positive control to confirm the secretory capacity of the islets. It is noteworthy that during the wash-out period at 90–130 min, the effect on ISV showed some “tale effect”, that is, the effect of ISV did not vanish immediately but declined gradually toward a basic level. This may be because ISV influences a receptor (e.g., a TRPM5-related receptor) and remains bound for some time, resulting in the effect gradually disappearing. TRPM5, transient receptor potential cation channel subfamily melastatin member 5, is a monovalent cation channel located in various human cells, including Type II taste receptor cells and pancreatic β-cells [48,49,50]. An increase in intracellular calcium would activate TRPM5; Philippaert K. et al. (2017) proved that the potentiation of the channel’s activity by steviol glycosides modulates taste responses and insulin release, which would explain the feature of the compound being sweet and lowering blood glucose concurrently [51].

**Figure 8:**
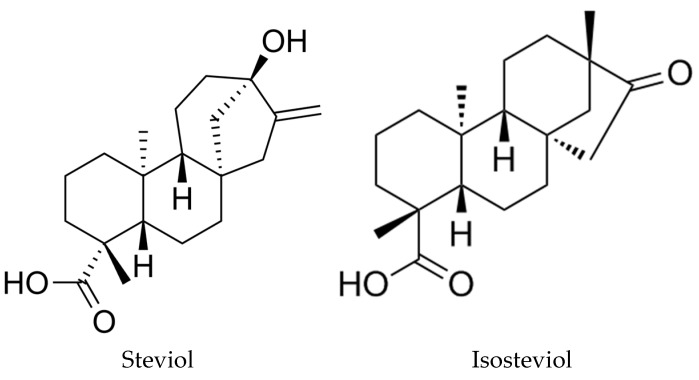
Chemical structure of steviol and isosteviol.

Steviol glycosides share a common aglycone core structure steviol (*ent*-13-hydrozykaur-16-en-18-oic acid), which as mentioned could be converted to ISV through acid hydrolysis. The chemical structures of steviol and ISV are very similar, see Figure 8, indicating that their insulin-secreting effect is mostly related to their common diterpene skeleton [52].

As illustrated in Figure 2, Figure 3 and Figure 4, the toxic concentrations of glucose (30 mM), palmitate (0.5 mM and 1 mM) or leucine (10 mM) caused INS-1E cell death, with an increasing toxic sequence: 10 mM leucine, 30 mM glucose, and 0.5 (or 1) mM palmitic acid. ISV did not counteract the detrimental effects caused by gluco-, lipo-, or aminoacidotoxicity. However, in the control groups the presence of ISV did not change cell viability, indicating the absence of cytotoxic effects of ISV and pointing to a promising safety profile of ISV.

The insulin secretion results depicted in Figure 5, Figure 6 and Figure 7 are in line with the results from cell viability studies. When INS-1E cells were exposed to gluco- or lipotoxicity, a large portion of cells were dead and undoubtedly not functioning well. Therefore, the insulin amount released from the cells was decreased dramatically. Surprisingly, in the present study the aminoacidotoxicity was minimal since cell death was only slightly increased and insulin secretion was unaffected during high amino acid levels.

Our results suggest that ISV possesses no protective effects on INS-1E cells when exposed to gluco-, lipo-, or aminotoxicity. Interestingly, we have previously found that after nine weeks of treatment with standard chow diet plus ISV, plasma glucose was reduced by 38% in KKAy diabetic mice, of which the plasma glucose levels were about 26 mM before the treatment [33]. The apparent discrepancy between the results from the two studies may indicate that the protective effects of ISV may not operate directly via an effect on INS-1E cells. Alternatively, INS1-E cells per se may not be sensitive to the protective effects of ISV after long-term gluco-, lipo, or aminoacidotoxicity.

We also included glucagon-like peptide-1- (7-36) amide (GLP-1) in this study to compare the pharmacological effects of these two compounds. GLP-1, a potent incretin hormone, has been developed into an important drug for the treatment of T2D [52]. Until now, few studies have investigated the effect of GLP-1 in α-cells regarding change in glucagon secretion and cell proliferation under gluco-, lipo-, and aminoacidotoxicity conditions, and no previous studies have compared its effect with ISV. GLP-1 and ISV seem to share a few features, e.g., both show glucose-dependent insulinotropic effects and both lower body weight. Like ISV, GLP-1 did not show significant effects on cell viability or insulin secretion during long-term gluco-, lipo-, or aminoacidotoxiciy in INS-1 cells.

Impressively, when INS-1E cells were exposed to low glucose (5.5 mM) there was no significant influence on cell viability, whereas the insulin secretion was dramatically raised. This may reflect that the cells are more sensitive to glucose stimulation when the prevailing glucose level is relatively low. This underlines the importance of maintaining optimal glycemic control.

## 6. Conclusions 

In conclusion, ISV did not increase cell death and, in this respect, it appears safe. We showed a pronounced dynamic effect of ISV on glucose-stimulated insulin secretion from mouse pancreatic islets. However, ISV does not counteract gluco-, lipo-, or aminoacidotoxicity in INS-1E cells. Further studies are required to demonstrate the antidiabetic effects of ISV and to further confirm its safety profile in humans.
